# Femoral hydatid cyst: A rare localization of bone echinococcosis: A case report

**DOI:** 10.1016/j.radcr.2024.12.035

**Published:** 2025-01-07

**Authors:** Nadia El Mahi, Hajar Siouri, Amal Mojahid, Karim Haddar, Hamid Ziani, Siham Nasri, Imane Kamaoui, Imane Skiker

**Affiliations:** Department of Radiology, Mohammed VI University Hospital, Faculty of Medicine, University Mohammed First, Oujda, Morocco

**Keywords:** Hydatid cyst, Echinococcosis, Bone localization, Femur, Imaging

## Abstract

Echinococcosis, or hydatid disease, is an endemic disease that affects many regions worldwide and remains a significant public health issue in areas with high endemicity. It is caused by an infection with the dog tapeworm *Echinococcus granulosus*, which is transmitted to humans either through direct contact with dogs or by ingesting contaminated food. This disease primarily affects internal organs, particularly the liver and lungs. Although virtually all organ systems can be involved, bone involvement is possible, especially in the femur. This report presents a case of an extensive hydatid cyst in the femoral bone of a 43-year-old woman, diagnosed late, highlighting the silent nature of bone echinococcosis due to its long latency period. Radiological examinations play a crucial role in the diagnosis, particularly when the disease is detected at an advanced stage. This case emphasizes the importance of early detection through imaging to allow for appropriate management and prevent complications.

## Introduction

Echinococcosis is an endemic zoonotic disease, common in countries where cattle farming and agriculture play a major role, particularly in Morocco and Mediterranean countries. The mortality associated with this disease ranges from 0.9% to 3.6% [1]. The causative agent is most often *E. granulosus*, with *E. multilocularis* being more rare. Humans act as intermediate hosts by coming into contact with a definitive host (usually a domestic dog) or by ingesting contaminated water or vegetables [2]. Approximately 60 to 70% of hydatid cysts form in the liver, while 10% to 15% affect the lungs [3]. Bone involvement in hydatid disease is relatively rare, representing about 0.5% to 2.5% of human hydatidosis cases [4]. This rarity of bone involvement is one of the reasons why diagnosis may be delayed, as bone echinococcosis is sometimes mistaken for other more common pathologies, such as bone infections or tumors. Previously referred to as “white cancer of the bone,” it carries a poor prognosis. The clinical manifestations of echinococcosis are variable and often absent for extended periods, making the disease difficult to detect until it reaches an advanced stage, at which point radiological lesions are widely spread [5]. Symptoms generally appear when the cysts increase in size, leading to pain, deformities, or pathological fractures. We report here the case of a 43-year-old female patient with extensive bone involvement of hydatid disease affecting the femoral bone, without any primary lesions in the liver. The diagnosis of intraosseous hydatid cyst was initially established through imaging and then confirmed by histopathological examination. The patient was treated with antiparasitic medication followed by surgical intervention.

## Case presentation

**Fig. 1:**
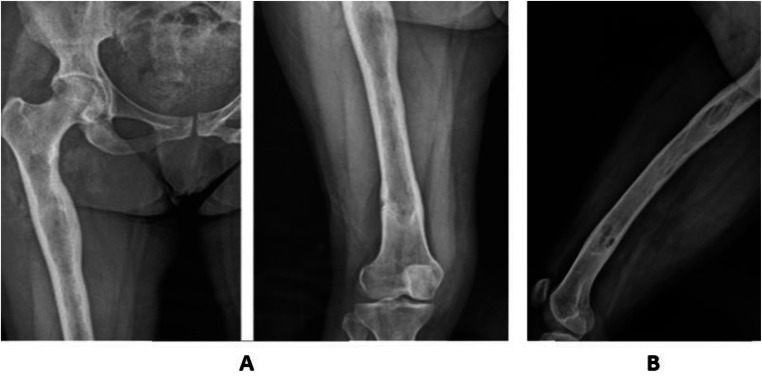
Standard Radiograph : F(A) /P (B) of the right lower limb. Extensive diaphyseal widening of the femoral bone associated with thickening of the cortical bone.

We present the case of a 43-year-old woman from a rural area who had been experiencing mechanical pain in her right hip radiating to the thigh and knee for the past 6 years. Initially treated as lumbar sciatica, her condition showed no clinical improvement. The pain started off mild but progressively worsened over the past 4 months, becoming more intense and significantly limiting her walking capacity. On physical examination, there was swelling and tenderness in the right thigh, but no signs of fever, anorexia, or weight loss were observed. Blood tests, including C-reactive protein (CRP) and white blood cell count, were within normal limits. Standard radiographs (Fig. 1) revealed significant diaphyseal widening of the femoral bone along with cortical thickening.

**Fig. 2:**
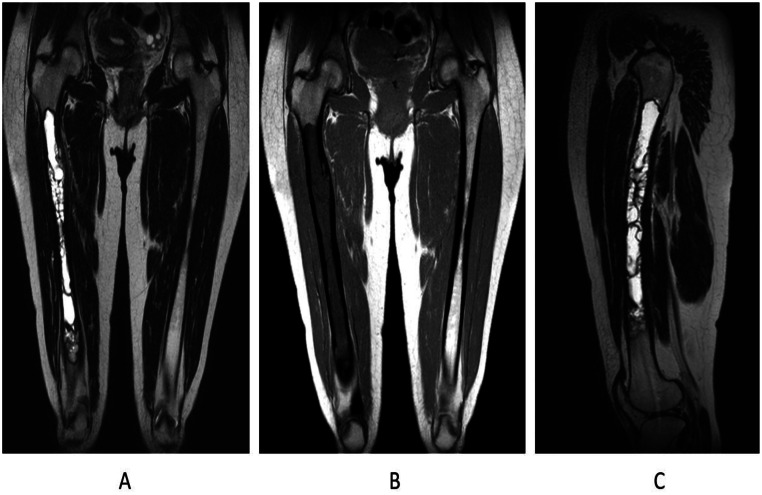
MRI of the right lower limb in coronal section T2 (A), coronal T1 (B) and sagittal section T2 (C): Centromedullary lesional process in the right femoral diaphyseal (25 cm of height) composed of many cystic formations, in low signal T1 and high signal T2, not enhanced after contrast.

**Fig. 3:**
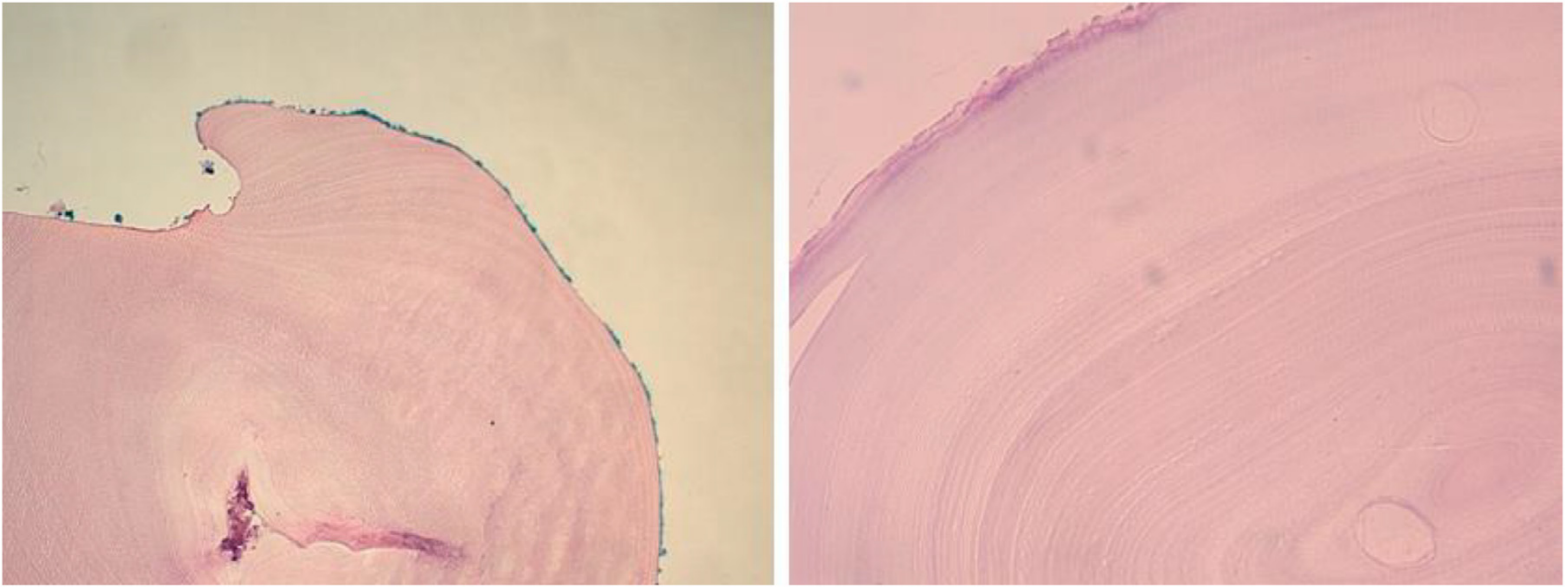
Histological study confirms the diagnosis of intraosseous hydatid cyst and shows the hydatid cuticle with an anhistic acidophilic appearance, lined with more or less detached unicellular proligeral membrane, with no visible scolex (H&E, 200).

Magnetic resonance imaging (MRI) of the thigh (Fig. 2) showed a large centromedullary lesion in the right femoral diaphysis, measuring 25 cm in length. The lesion was characterized by multiple cystic formations, which were hyperintense on T2-weighted images, hypointense on T1-weighted images, and did not enhance postcontrast. This multi-vesicular appearance was highly suggestive of intraosseous hydatid cysts. A bone biopsy was performed, and histopathological examination (Fig. 3) confirmed the diagnosis of an infected intraosseous hydatid cyst. A microbiological culture was also taken, and PCR testing for Mycobacterium tuberculosis complex DNA was negative. Following histological confirmation, the patient was started on an antiparasitic treatment regimen with albendazole 400 mg twice daily, in addition to an antibiotic therapy consisting of Oroken 200 mg twice daily and Catex 1 mg 3 times daily. Three months later, the patient underwent surgical intervention for centromedullary lavage of the hydatid cyst. The immediate postoperative course was uneventful, and albendazole treatment was continued for 6 weeks to prevent recurrence. The patient then underwent a full radiological workup, including abdominal ultrasound and CT scans of the brain, spine, thorax, and abdomen, to rule out any hepatic or pulmonary involvement. The results confirmed that the hydatid disease was limited solely to the femoral bone, with no other sites of infection. Unfortunately, the patient was lost to follow-up and did not undergo postoperative imaging to assess disease progression.

## Discussion

The diagnosis of bone echinococcosis is challenging and primarily relies on the endemic region, clinical findings, radiological imaging, and serological tests [5]. Humans, as accidental intermediate hosts [6], typically become infected in sheep farming areas through direct contact with dogs or indirect ingestion of contaminated food or water. Shepherds, veterinarians, and children are particularly at risk. Contamination can also occur through the ingestion of raw vegetables contaminated by dog feces that are inadequately washed [7], as was the case with our patient, who lived in a rural environment. While the liver and lungs are the most commonly affected organs, echinococcosis can also involve the bones, particularly the long bones, which account for approximately 30% of bone-localized cases. Femoral involvement represents around 10% of these cases [8]. Other bone sites commonly reported in the literature include the vertebrae, pelvis, and hip [,^1^,^9^,^10^]. Rare cases have also been documented in the ribs and humerus. The clinical presentation of the disease is often delayed, as the condition remains asymptomatic for an extended period. This slow progression is one reason why the diagnosis is often made late, typically after a pathological fracture, secondary infection, or vertebral lesions resulting in compressive myelopathy [11].

The diagnosis of bone hydatidosis mainly relies on radiographic imaging. Initial lesions in long bones are usually located in the metaphysis or epiphysis, and in more advanced stages, they can extend to the diaphysis, which appears widened [5]. Conventional radiography remains one of the most effective diagnostic tools for bone hydatid lesions, with the most common features being rounded or oval osteolytic lesions, often multiple and confluent, with clear sclerotic borders and a cystic appearance. The diameter of these lesions can vary and sometimes leads to bone expansion with cortical involvement. Occasionally, osteolytic areas with poorly defined margins are observed, associated with pathological fractures and extension to adjacent soft tissues, mimicking a tumoral process [12]. Magnetic resonance imaging (MRI) is considered the gold standard imaging technique due to its high contrast resolution and 3-dimensional assessment capabilities, which provide better evaluation of both local and regional spread, as well as involvement of soft tissues. MRI is also crucial for detecting early recurrences of hydatid cysts after treatment. Hydatid cysts typically show hypointensity on T1-weighted images and hyperintensity on T2-weighted images. The MRI findings in our case were consistent with those reported in the literature [5].

Histological examination of the surgical specimen is essential and remains the definitive diagnostic tool. The optimal therapeutic approach combines both surgical and medical treatments. Various authors have proposed different strategies, particularly regarding the duration of antiparasitic therapy following surgical excision. The majority of studies recommend surgical intervention followed by a 6-month course of Albendazole at a dose of 15 mg/kg/day orally [13], which was the treatment protocol used for our patient.

## Conclusion

Bone echinococcosis is a rare condition, but it represents a significant public health issue, particularly in endemic areas such as Morocco. This insidious disease is characterized by diagnostic challenges due to its often late and nonspecific clinical presentation. Radiological imaging plays a crucial role in diagnosing hydatidosis, which is typically detected at an advanced stage of the disease. However, the definitive diagnosis is based on histopathological results. The optimal therapeutic strategy combines both medical and surgical treatments.This case report highlights the importance of early detection and prompt, appropriate management of bone echinococcosis to improve patient outcomes.

## Declaration of generative AI and AI-assisted technologies in the writing process

During the preparation of this work the author(s) used ChatGPT in order to improve language and readability; After using this tool/service, the author(s) reviewed and edited the content as needed and take(s) full responsibility for the content of the publication.

## Patient consent

An informed consent was obtained from the patient.
